# Synaptic Activity in Serotonergic Neurons Is Required for Air-Puff Stimulated Flight in *Drosophila melanogaster*

**DOI:** 10.1371/journal.pone.0046405

**Published:** 2012-09-27

**Authors:** Sufia Sadaf, Serge Birman, Gaiti Hasan

**Affiliations:** 1 National Centre for Biological Sciences, Tata Institute of Fundamental Research, Bangalore, Karnataka, India; 2 Laboratoire de Neurobiologie, Centre National de la Recherche Scientifique, Ecole Supérieure de Physique et de Chimie Industrielles, ParisTech, Paris, France; Center for Genomic Regulation, Spain

## Abstract

**Background:**

Flight is an integral component of many complex behavioral patterns in insects. The giant fiber circuit has been well studied in several insects including Drosophila. However, components of the insect flight circuit that respond to an air-puff stimulus and comprise the flight central pattern generator are poorly defined. Aminergic neurons have been implicated in locust, moth and Drosophila flight. Here we have investigated the requirement of neuronal activity in serotonergic neurons, during development and in adults, on air-puff induced flight in Drosophila.

**Methodology/Principal Findings:**

To target serotonergic neurons specifically, a Drosophila strain that contains regulatory regions from the *TRH* (Tryptophan Hydroxylase) gene linked to the yeast transcription factor GAL4 was used. By blocking synaptic transmission from serotonergic neurons with a tetanus toxin transgene or by hyperpolarisation with Kir2.1, close to 50% adults became flightless. Temporal expression of a temperature sensitive Dynamin mutant transgene (*Shi^ts^*) suggests that synaptic function in serotonergic neurons is required both during development and in adults. Depletion of IP_3_R in serotonergic neurons via RNAi did not affect flight. Interestingly, at all stages a partial requirement for synaptic activity in serotonergic neurons was observed. The status of serotonergic neurons was investigated in the central nervous system of larvae and adults expressing tetanus toxin. A small but significant reduction was observed in serotonergic cell number in adult second thoracic segments from flightless tetanus toxin expressing animals.

**Conclusions:**

These studies show that loss of synaptic activity in serotonergic neurons causes a flight deficit. The temporal focus of the flight deficit is during pupal development and in adults. The cause of the flight deficit is likely to be loss of neurons and reduced synaptic function. Based on the partial phenotypes, serotonergic neurons appear to be modulatory, rather than an intrinsic part of the flight circuit.

## Introduction

Animal behavior is a complex stimulus-driven process that requires coordinated interaction between specific neural circuits. Neuronal activity has been shown to play an important role in the development, maintenance and modulation of these circuits [1], [2]. Animals exhibiting simple behaviors have often been used to understand mechanisms underlying neural circuit development and function [3]. Our interest is to identify individual components of the neural circuits required for insect flight, through a genetic and cellular approach in the fruit fly, *Drosophila melanogaster*.

Triggering of flight by the giant-fiber mediated escape response pathway (also called the giant fiber pathway) has been relatively well studied in Drosophila [4], [5], [6], [7]. Escape response pathways are activated under conditions perceived as a threat by the animal, such as a bright flash of light. The organization of these circuits is usually less complex because speed of response is critical for survival. Insect flight can also be initiated by non-threatening stimuli like a gentle puff of air. Air-puff stimulated flight is thought to be mediated by an alternate pathway [5], [8]. A requirement for the biogenic amines, octopamine and tyramine in modulation of insect flight has been shown from studies in locusts, Manduca and other moths [9], [10], [11]. More recently, using an octopaminergic neuronal driver, *dTdc-2GAL4*, octopamine has been shown to play a modulatory role in Drosophila flight [12]. Although the neural components of air-puff stimulated flight, measured in tethered flies, remain largely unknown, previous studies have shown that serotonergic and dopaminergic neurons, which are another subset of aminergic neurons, could play a role in the function/modulation of this circuit [8].

Fly mutants of the inositol 1,4,5 trisphosphate receptor (IP_3_R) gene, *itpr*, are unable to evoke air-puff stimulated flight, even though physiological responses on stimulation of the giant-fiber pathway remain unaltered. Previous work has demonstrated that expression of an *itpr^+^* cDNA in aminergic neurons (using the *DdcGAL4* driver) rescued loss of flight in *itpr* mutants close to wild-type levels and blocking of synaptic activity in aminergic neurons by tetanus toxin expression reduced flight to 45% [8]. Moreover, an adult requirement for the serotonergic component of aminergic neurons was indicated, since a flight deficit of 33% was observed in wild-type adult flies fed for 5 days with a serotonin synthesis inhibitor, para-chlorophenylalanine (PCPA) [8]. Thus a role for synaptic activity in aminergic neurons was indicated, with a possible requirement for serotonin both during development and in adult flight. More recently, it was shown that intracellular Ca^2+^ signaling through IP_3_R and store-operated Ca^2+^ entry (SOCE) in neurons are important for air-puff induced flight [13], suggesting that aminergic neuron function in Drosophila flight might require IP_3_R mediated Ca^2+^ signals.

In this study, we have studied the effect of blocking synaptic function and reduced intracellular Ca^2+^ signaling specifically in serotonergic neurons, on air-puff stimulated flight. It is known that the insect flight circuit is formed during pupal development [8], [14], [15], [16]. Therefore, the effect of blocking synaptic activity in serotonergic neurons during pupal development and in adults was assessed. We show that blocking synaptic activity in serotonergic neurons either during flight circuit development or in adults reduces air-puff induced flight significantly. Our data suggest that synaptic activity affects the number of flight modulating serotonergic neurons in the second thoracic segment, but modulation of flight by these neurons does not require the IP_3_R or SOCE.

## Materials and Methods

### Fly Stocks

Driver: *TRHGAL4*, with regulatory region of the *Tr*yptophan *H*ydroxylase gene present upstream of yeast GAL4; expression in serotonergic neurons (from S. Birman's laboratory, unpublished). UAS effector genes: *UASTNTH* (gene for active L-chain of tetanus toxin, tnt) [17], *UASTNTvif* (inactive tetanus toxin), *UASKir2.1* (gene for human K^+^ inward rectifier channel, isolated from human cardiac cells) from Bloomington Stock Centre, Bloomington, IN, USA [18], *UASShi^ts^* from Toshi Kitamoto (University of Iowa, Iowa City, IA, USA) [19]. *UASRNAi* strains for *dOrai* and *dSTIM* were obtained from the Vienna Drosophila RNAi Centre, Vienna, Austria [20] and for *itpr* from the National Institute of Genetics Fly Stocks Centre, Kyoto, Japan. *UASmCD8GFP* (Bloomington Stock Centre, Bloomington, IN) was used to mark neurons. A recombinant strain, *TRHGAL4, UASmCD8GFP* was generated using standard fly genetics protocol for visualization of serotonergic neurons.

### Flight assay

Flight tests were performed using modified cylinder drop assay as previously described [8]. Flies were collected in batches of 20 (on ice) just after eclosion and were aged for 3 days at 25°C, unless mentioned otherwise. These batches were dropped into a 1 m long glass cylinder. Flies that fell through directly into a chilled conical flask were scored as non-fliers and those that flew and sat on the walls of the cylinder were scored as fliers. Computation of means and SEMs were performed on results obtained from at least 100 flies, using Origin 7.5 software (MicroCal, Origin Lab, Northampton, MA, USA) and statistical significance was determined by Student's *t* test for independent populations, p<0.05.

### Temperature shift experiments

Genetic crosses were set up with *Shi^ts^* lines at the permissive temperature of 25°C. White pupae (0 hr) were shifted to the non-permissive temperature (29°C) until eclosion. Animals were further aged for three days at 29°C, till they were tested for flight. For adult specific experiments, 2 day old flies were shifted to 29°C and kept at this non-permissive temperature for one day and thereafter tested for flight. For electrophysiological experiments, tethered flies were maintained in a moist chamber at 29°C for 1 hr before the actual recordings, which were carried out as rapidly as possible at room temperature (approximately 25°C).

### Electrophysiological recordings

Flies were anaesthetized on ice for 15 min and glued to a thin metal wire between the neck and the thorax with nail polish [8]. To record air puff responses, a gentle mouth-blown air puff stimulus was delivered to the fly kept in a tethered condition and movie was recorded for 30 s. Physiological recordings were performed on DLMs of the GF pathway. After recovery from anesthesia, an un-insulated 0.127 mm tungsten electrode, whose tip was sharpened by electrolysis to attain a tip diameter of 0.5 µm, was carefully inserted in the DLM (fiber a), just beneath the cuticle. A similar electrode was inserted in the abdomen as reference. Spontaneous firing was recorded for 2 min and air puff-induced recordings were performed for 30 s. All recordings were made using an ISODAM8A (World Precision Instruments, Sarasota, Florida, USA) amplifier with filter set up for 30 Hz (low pass) to >10 kHz (high pass). Gap-free mode of pClamp8 (Molecular Devices, Sunnyvale, CA, USA) was used to digitize the data (10 kHz) on a Pentium 5 computer equipped with Digidata 1322A (Molecular Devices). Data were analyzed using Clampfit (Molecular Devices) and plotted using Origin 7.5 software (MicroCal). Response to air puff and spontaneous activity were recorded from 10 or more flies for every genotype.

### Immunohistochemistry

Immunohistochemistry was performed on Drosophila adult brains, expressing a membrane bound GFP (*UASmCD8GFP*) with *TRHGAL4*, after fixing the dissected tissue in 4% paraformaldehyde. Flies were aged for three days, fliers and non-fliers were separated in a column flight test. Dissection was carried on the 4^th^ day. The following primary antibodies were used: mouse monoclonal anti-5-HT (1∶50; #MS1431S, NeoMarkers, Fremont, CA, USA), rabbit anti-GFP antibody (1∶10,000; #A6455, Molecular Probes, Eugene, OR, USA). Fluorescent secondary antibodies were used at a dilution of 1∶400 as follows: anti-rabbit Alexa Fluor 488 (#A1108) and anti-mouse Alexa Fluor 568 (#A1104) (Molecular Probes). Confocal analysis was performed on an Olympus Confocal FV1000 microscope using either a 20× objective with a numerical aperture (NA) of 0.9 or a 63× objective with 1.4 NA. Confocal data were acquired as image stacks of separate channels and combined and visualized as three-dimensional projections using the FV10-ASW 1.3 viewer (Olympus Corporation, Tokyo, Japan).

## Results

### Reduced flight ability due to loss of synaptic activity in serotonergic neurons

**Figure 1:**
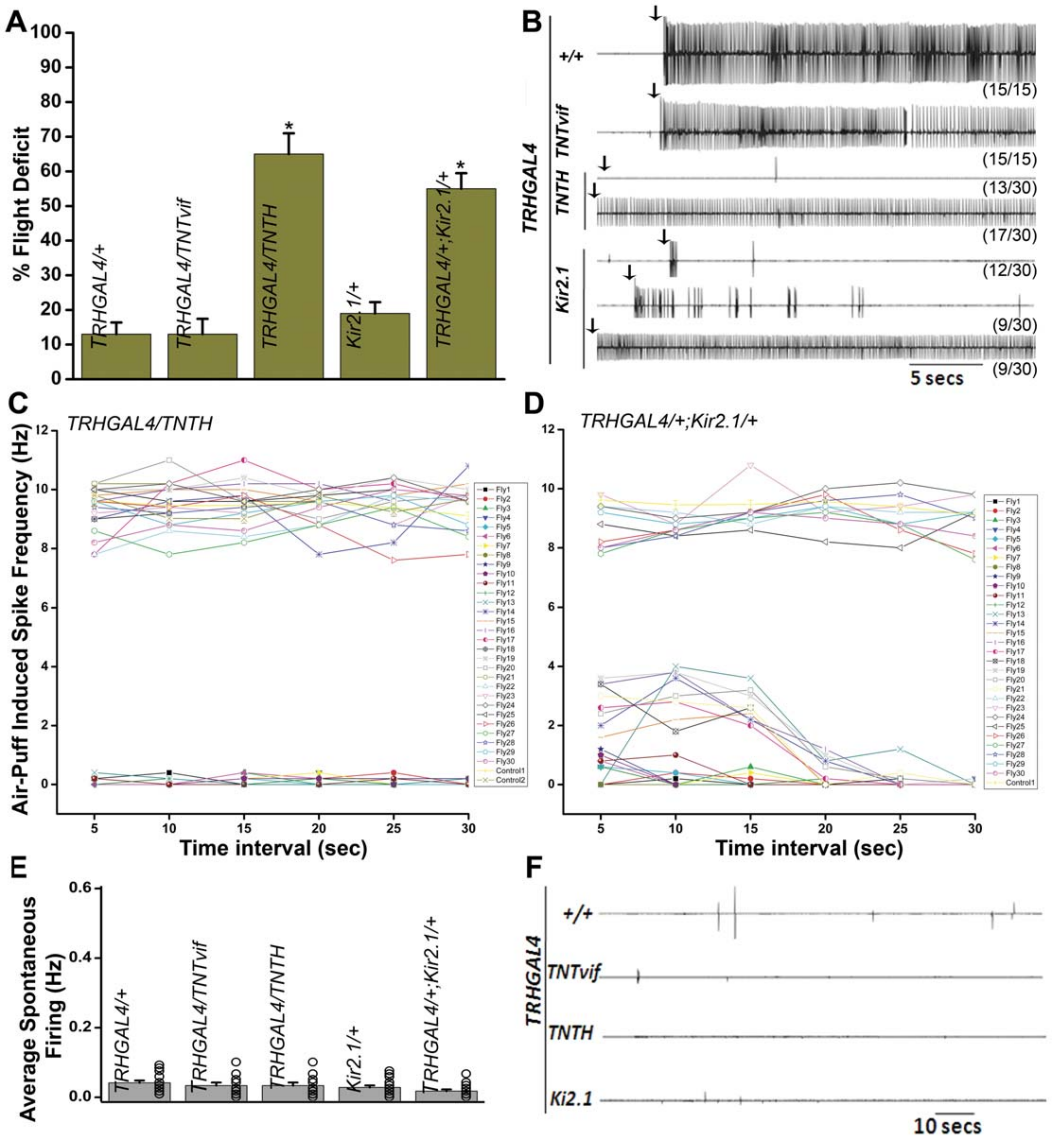
Loss of synaptic activity in serotonergic neurons causes flight defects. **A**) Flight deficit, assayed by the cylinder drop test, is significantly higher in animals expressing either tetanus toxin (TNTH) or the hyperpolarizing K^+^ ion channel (Kir2.1) as compared with controls (*p<0.005; Student's *t* test). Approximately 100 or more flies were tested for each genotype. Results are expressed as mean ± SEM. **B**) Electrophysiological recordings from the DLMs of tethered flies after delivery of an air puff stimulus (arrows). Control flies show rhythmic firing throughout flight. Loss of electrical activity is seen in 13/30 animals expressing *TNTH*. The remaining animals show wild-type like flight pattern. The duration of flight is reduced to <5 secs in 12/30 flies expressing *Kir2.1*. Intermittent flight patterns are seen in 9/30 flies. The remaining flies show wild-type like flight pattern. **C**) Quantification of the spike frequency during flight at a bin interval of 5 secs. Control flies (*TRHGAL4/+*, control 1 and *TRHGAL4/TNTvif*, control 2) show a spike frequency of 9 Hz in all the bins. The trace is expressed as an average of 15 flies. *TNTH* expressing flies show either complete loss of flight or normal flight frequency. **D**) Control flies (*Kir2.1/+*) show an average spike frequency of 9 Hz (15 flies). Flies expressing *Kir2.1* show variable spike frequencies. Expression of either TNTH or Kir2.1 in serotonergic neurons does not affect the frequency of spontaneous firing as recorded from the DLMs. **E**) Quantification of spontaneous firing. **F**) Representative traces of electrophysiological recordings from the DLMs.

Previously, the role of evoked synaptic activity in aminergic neurons has been studied in the context of flight, by expressing tetanus toxin in the aminergic domain [8] with *DdcGAL4*, which expresses in both dopaminergic and serotonergic neurons [21]. Flight ability in a cylinder drop test was reduced to 45% in these animals. However, the contribution of individual dopaminergic and serotonergic domains has not been assessed in flight circuit development and function. To investigate the role of serotonergic neurons in flight, a *TRHGAL4* strain that expresses in a majority of serotonergic neurons was used to drive expression of either active tetanus toxin, TNTH (with *UASTNTH*) or Kir2.1 (*UASKir2.1*). Expression of *TNTH* in neurons can abolish evoked synaptic activity by enzymatic cleavage of the synaptic vesicle associated protein, n-Synaptobrevin [17], whereas expression of an inward rectifying Kir2.1 channel causes voltage-dependent K^+^ ion channel opening, which shunts the membrane voltage toward the equilibrium potential of K^+^, thereby hyperpolarizing the neuronal membrane and making it refractory to synaptic activity [18], [22]. The resulting animals were first assayed for flight in the cylinder drop test. Flight deficits were observed with both TNTH and Kir2.1 expression (65%±6 and 55%±4.5 respectively; Fig. 1A). Flies expressing an inactive form of tetanus toxin (*UASTNTvif*) in serotonergic neurons did not show flight deficits that were significantly different from controls (*TRHGAL4/+*).

Next, air-puff stimulated flight responses were recorded from the dorsal longitudinal indirect flight muscles (DLMs) of single flies. In 13/30 flies expressing TNTH in TRH neurons there was no response to an air-puff, while 17/30 flies showed sustained rhythmic flight patterns similar to controls. Kir2.1 expression affected flight to varying degrees. In 12/30 flies, flight duration was reduced to <5 sec, i.e., the animals could initiate flight briefly. Intermittent flight was observed in 9/30 flies. The remaining flies showed wild-type flight patterns (Fig. 1B). Spike frequencies were calculated over 5 s bin intervals for all the categories. In 13/30 flies expressing TNT, the spike frequency was zero, while the remaining flies showed wild-type like frequencies throughout (Fig. 1C). In 12/30 flies expressing Kir2.1, the spike frequency was 1 Hz at initiation and then dropped to zero in all subsequent intervals. Flies that showed intermittent flight mostly initiated flight with a frequency of 2–4 Hz that lasted for 10–15 sec and then dropped to zero. The remaining flies showed wild-type like frequencies throughout (8–10 Hz) (Fig. 1D). Spontaneous synaptic activity recorded from the DLMs in the absence of any stimulus was not altered in any of the genotypes (Fig. 1E, F). Wing posture and morphology and performance in the climbing test were similar to control flies (data not shown). Thus, evoked synaptic activity from serotonergic neurons can affect normal flight to a significant extent. However, loss of serotonin release does not lead to increased spontaneous activity.

### Synaptic function in serotonergic neurons is required during pupal development and in adults

**Figure 2:**
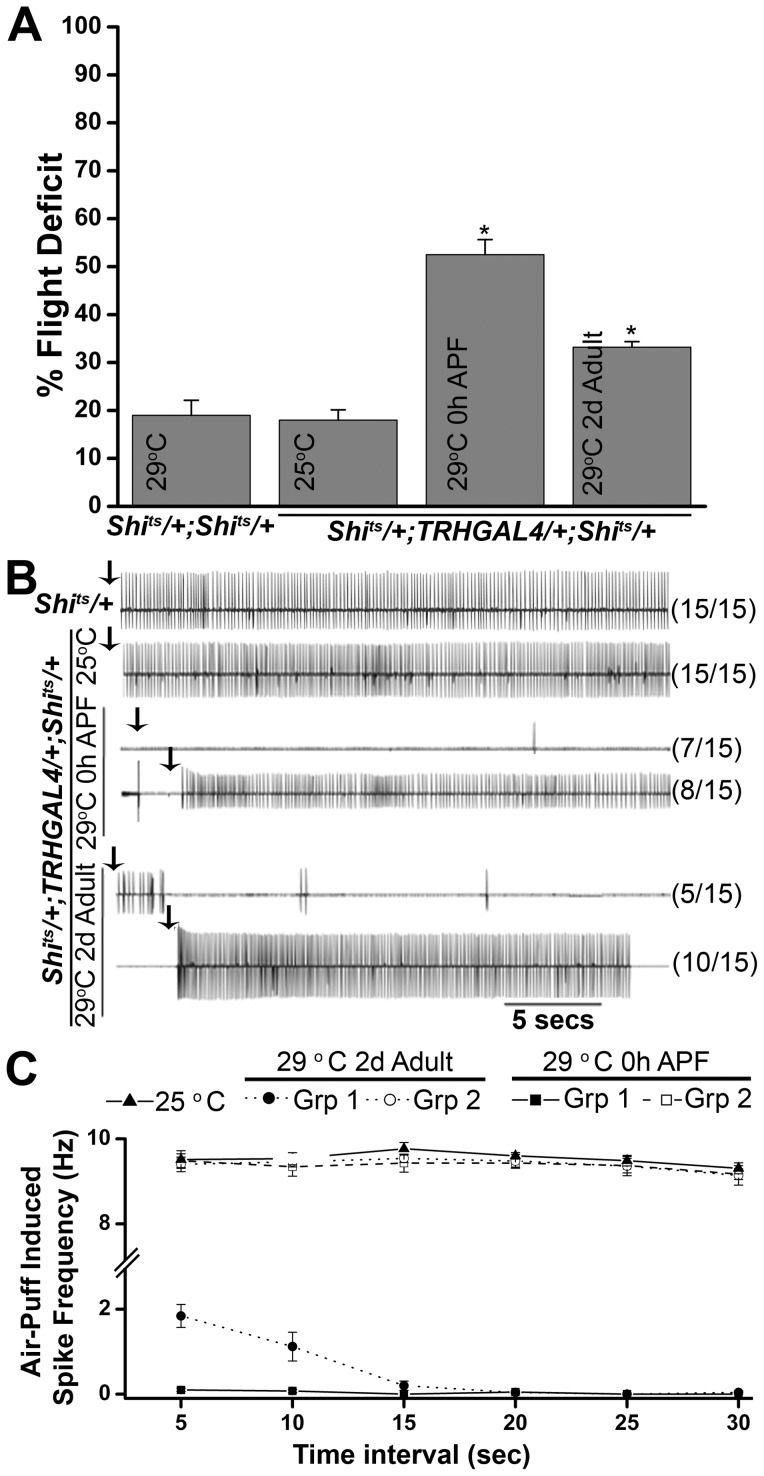
Synaptic activity in serotonergic neurons is required both during pupal development and in adults for flight. **A**) Flies with Shi^ts^ expression in serotonergic neurons throughout pupal development (0 h after puparium formation, APF) exhibit a 50% flight deficit in the column test. A lesser but significant deficit is also seen in flies expressing Shi^ts^ 2 days post eclosion. **B**) Air-puff stimulated flight response from the DLMs. Control flies, expressing Shi^ts^ in dopaminergic neurons and maintained at 25°C show rhythmic flight patterns. Animals expressing Shi^ts^ 2 days post-eclosion can initiate flight (5/15). Remaining flies show wild-type flight patterns. Shi^ts^ expression throughout pupal development causes complete loss of electrical activity in 8/15 flies. Remaining flies show wild-type flight patterns. **C**) Quantification of air-puff stimulated spike frequency. The traces are presented as an average of the indicated numbers. Control flies expressing Shi^ts^ at the permissive temperature (25°C) show a spike frequency of 9 Hz (15 flies). Shi^ts^ expression at the non-permissive temperature (29°C) during pupal development shows complete loss of spikes in all the intervals in 8/15 flies (group 1), while the remaining flies (group 2) show spike patterns like the controls. When flies expressing Shi^ts^ are maintained at 29°C from 2-days post eclosion, the spike frequency at initiation remains low (2 Hz) and then diminishes further in 5/15 adults tested. The remaining flies (group 2) show wild-type like frequencies. Spontaneous firing remains unaffected (data not shown).

To study the temporal requirement for synaptic activity in serotonergic neurons, a temperature sensitive mutant of dynamin *Shibire^ts^* (*UASShi^ts^*) was expressed with *TRHGAL4* [19], [23]. Dynamin is a GTPase which is required for recycling of synaptic vesicles. Expression of Shi^ts^ at restrictive temperatures reduces endocytotic synaptic vesicle recycling, thereby reducing synaptic transmission [23]. Experimental animals were shifted to the non-permissive temperature (29°C) either at 0 hr pupae phase or 2 days post eclosion. Flight defects were observed in animals expressing Shi^ts^ both during pupal development or 2 days post eclosion (Fig. 2A). Flight defects were more severe when synaptic activity was blocked during pupal development (52.5%±3.14) than in animals expressing Shi^ts^ 2 days post eclosion (33.2%±1.2). Air-puff stimulated responses recorded from DLMs were absent in 7/15 flies expressing Shi^ts^ during pupal development (Fig. 2B). The remaining flies showed wild-type like flight patterns. When flies expressing Shi^ts^ in serotonergic neurons were shifted to the non-permissive temperature as 2d old adults, electrophysiological recording from the DLMs showed that 5/15 flies could initiate but were unable to maintain flight while the remaining flies showed normal flight patterns. The 5 flies that could not maintain flight showed low spike frequencies (∼2 Hz) for the first 5 sec followed by no spikes in subsequent bins while the remaining flies showed wild-type like flight frequencies (Fig. 2C). These data suggest that there is a greater requirement for synaptic activity in serotonergic neurons during flight circuit development, followed by reduced requirement in adults.

### Depletion of IP_3_R and SOCE in serotonergic neurons does not affect flight

**Figure 3:**
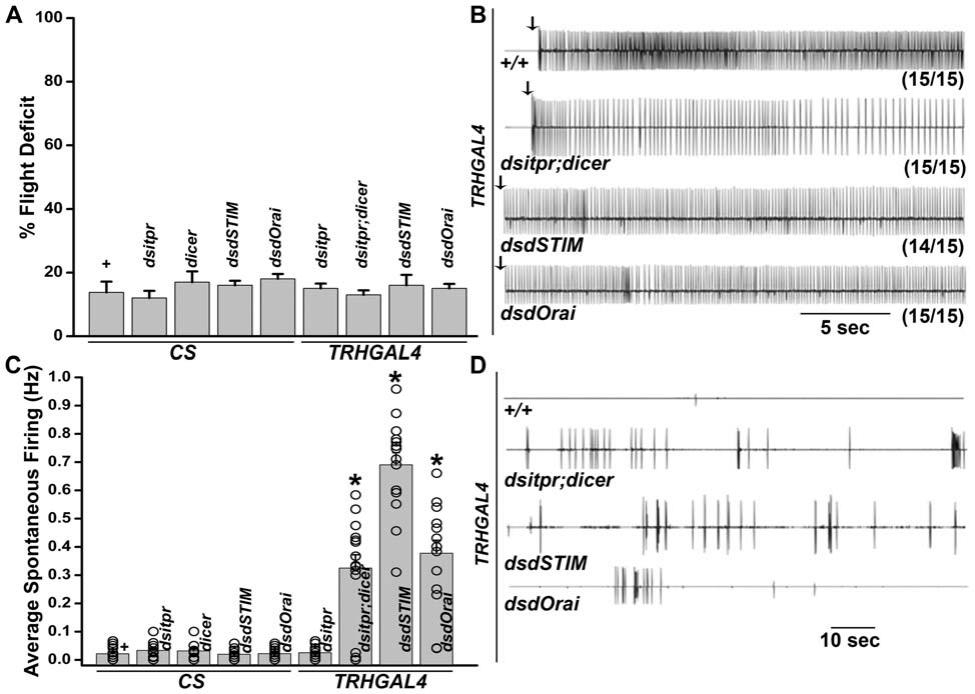
RNAi knock-down of of IP_3_R or SOCE components in serotonergic neurons does not affect flight. **A**) In the cylinder drop test, no flight defect is seen in flies expressing RNAi against IP_3_R, STIM or Orai as compared with controls. For each genotype, a total of 100 flies were tested in 5 batches of 20. **B**) Electrophysiological recordings from the DLMs of tethered flies after delivery of an air puff stimulus (arrows). All flies show rhythmic firing throughout flight. **C**) Quantification of spontaneous firing. Depletion of IP_3_R or SOCE increases spontaneous firing. (*p<0.05; Student's *t* test). **D**) Representative traces of electrophysiological recordings from the DLMs.

IP_3_R mutants in Drosophila are flightless and show increased spontaneous firing from the DLMs. Expression of the IP_3_R in dopaminergic and serotonergic neurons (a subset of aminergic neurons) by *DdcGAL4* is sufficient to restore flight to *itpr* mutants [8]. While, pan-neuronal knockdown of either IP_3_R or components of Store-operated calcium entry, STIM and Orai, resulted in flight defects similar to the *itpr* mutants [13], [24], aminergic neuron-specific knockdown (driven by *DdcGAL4*) did not show a flight phenotype, with the exception of increased spontaneous firing from the DLMs [8], [24]. To test the requirement of IP_3_R or SOCE function in serotonergic neurons specifically, *TRHGAL4* was used to drive the expression of previously tested RNAi knock-down constructs for *itpr*, *dSTIM* and *dOrai*. Similar to previous results with *DdcGAL4* driven knockdown, wing posture and flight defects were absent (Fig. 3A, B). However increased spontaneous firing from the DLMs was observed with all three RNAi lines when expressed in *TRH* neurons (Fig. 3C, D). Thus, perturbation of intracellular Ca^2+^ homeostasis in serotonergic neurons does not affect flight and suggests that evoked synaptic activity in these neurons is independent of intracellular Ca^2+^ signaling and SOCE.

### Inhibition of synaptic function affects number of serotonergic neurons in the second thoracic segment

**Figure 4:**
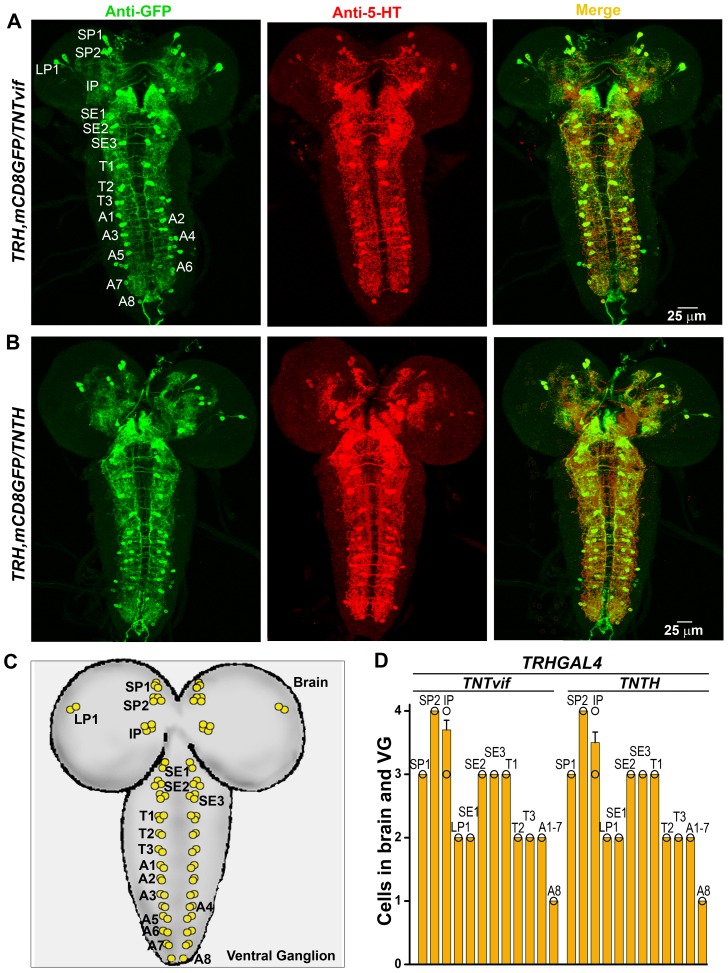
Loss of synaptic activity in serotonergic neurons does not affect the cell population in the larval central nervous system. **A**) Immunohistochemistry of the larval brain expressing *mCD8GFP/TNTvif* in *TRHGAL4* domains (control). All the GFP stained cells also show anti-5-HT staining (merge). **B**) Immunohistochemistry of the larval brain expressing *mCD8GFP/TNTH* in *TRHGAL4* domains. No difference is seen as compared with control. **C**) Schematic of *TRHGAL4/mCD8GFP* and 5-HT positive neurons marked in the larval brain. **D**) Number of cells marked by anti-GFP and anti-5-HT staining does not vary between control and tetanus toxin expressing animals.

To understand how inhibition of synaptic function in TRH neurons during pupal development affects flight, we visualized TRH positive neurons in TNT expressing flier and non-flier populations, and compared these with animals expressing inactive TNT (*UASTNTvif*). For this purpose a recombinant strain was generated expressing a membrane bound GFP (*UASmCD8GFP*) with *TRHGAL4.* Initially, third instar larval brains from animals expressing Tetanus toxin (*UASTNTH*) and control animals expressing inactive tetanus toxin (*UASTNTvif*) were visualized. These showed no significant difference in serotonergic cell populations as judged by anti-GFP and anti-5-hydroxytryptamine (5-HT, serotonin) immunostaining (Fig. 4A, B). The number of cells observed in each defined neural segment, were similar to earlier reports (Fig. 4C, D) [25], [26].

**Figure 5:**
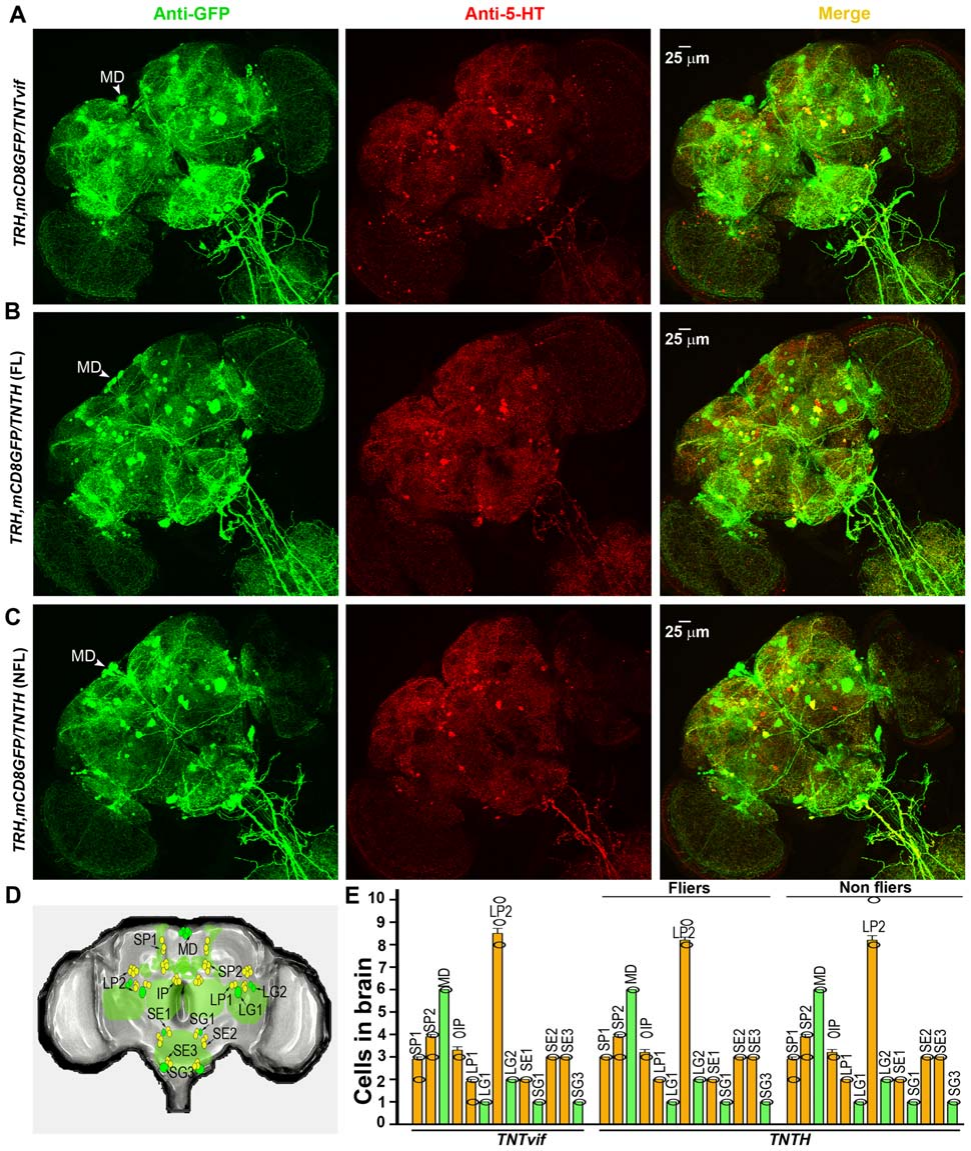
Loss of synaptic activity in serotonergic neurons does not affect cell numbers in the adult brain. **A**) Immunohistochemistry of the adult brain expressing *mCD8GFP/TNTvif* in *TRHGAL4* domains. All *TRHGAL4* positive neurons (anti-GFP, green) stain with anti-5-HT (red and green merge), except for 6 medial cells (MD), 1 cell in LP1 (LG1), 2 in LP2 (LG2), 1 in SE1 (SG1) and 1 in SE3 (SE3), which are GFP-positive but 5-HT negative. **B**) Immunohistochemistry on a *TRHGAL4/TNTH* brain collected from flies which passed the column flight test (fliers). Medial cells are seen (green). **C**) Immunohistochemistry on a *TRH/TNTH* brain collected from non-fliers. No difference is seen as compared with controls in A and B. **D**) Schematic of the brain showing cells marked by *TRHGAL4* (anti-GFP, green) and anti-5-HT. Medial cells are not stained with anti-5-HT. **E**) Number of cells marked by anti-GFP and anti-5-HT staining does not vary between control and tetanus toxin expressing serotonergic neurons among fliers and non-fliers. (Nomenclature based on [26]).

Next, numbers of serotonergic neurons were quantified in the central brain of adults expressing TNT or TNTvif (Fig. 5A–C). The numbers of previously identified 5-HT positive neurons (Fig. 5D), were no different in TNT expressing fliers and non-fliers as well as TNTvif controls (Fig. 5E). However, 6 GFP-positive medial cells, 1 cell in the Lp1 cluster, 2 cells in the LP2 cluster, 1 cell in SE1 and 1 cell in the SE3 clusters were observed in the brain which did not stain with anti-5-HT (Fig. 5A–E). These neurons were of a larger size as compared with other neurons. Similar non-5-HT positive medial cells have been observed in another *TRHGAL4* strain [27], implying that these neurons are TRH positive but don't synthesize 5-HT at detectable levels. Overall, there was no significant difference in the number of cells between controls and the brains of either fliers or non-fliers expressing TNT in TRH neurons (Fig. 5E).

**Figure 6:**
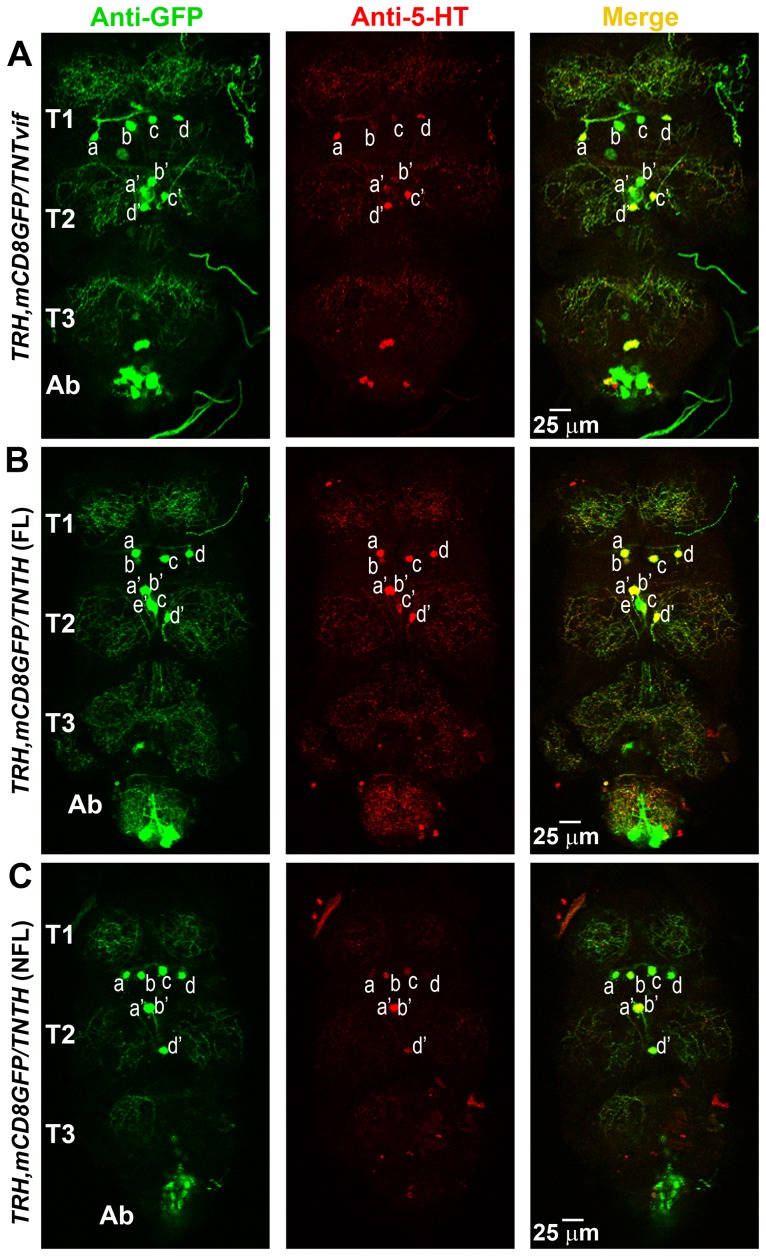
Loss of synaptic activity in serotonergic neurons reduces the cell numbers in thoracic ganglia. **A**) Immunohistochemistry of a thoracic ganglion expressing mCD8GFP and TNTvif in serotonergic neurons (sample S2, Fig. 7D). The thoracic segment has 4 cells (T1a–d) in T1 region and 4 cells (T2a′–d′) in the T2 region (anti-GFP, green). Anti-5-HT staining (red) also follows the same pattern. **B**) Immunohistochemistry on *TRH/TNTH* thoracic ganglia collected from flies which passed the column flight test (fliers). Anti-GFP staining shows 4 cells in T1 and 5 cells, T2a′–e′, in the T2 region. Anti-5-HT does not stain T2e′ (sample S6, Fig. 7C). **C**) Immunohistochemistry on *TRH/TNTH* thoracic ganglia collected from non-fliers. Anti-GFP staining shows 4 cells (T1a–d) in T1 and 3 cells (T2a′,b′,d′) in T2 region (sample S3, Fig. 7E). **D**) Schematic representation of serotonergic neurons as seen in T1 and T2 region of the thoracic ganglia.

**Figure 7:**
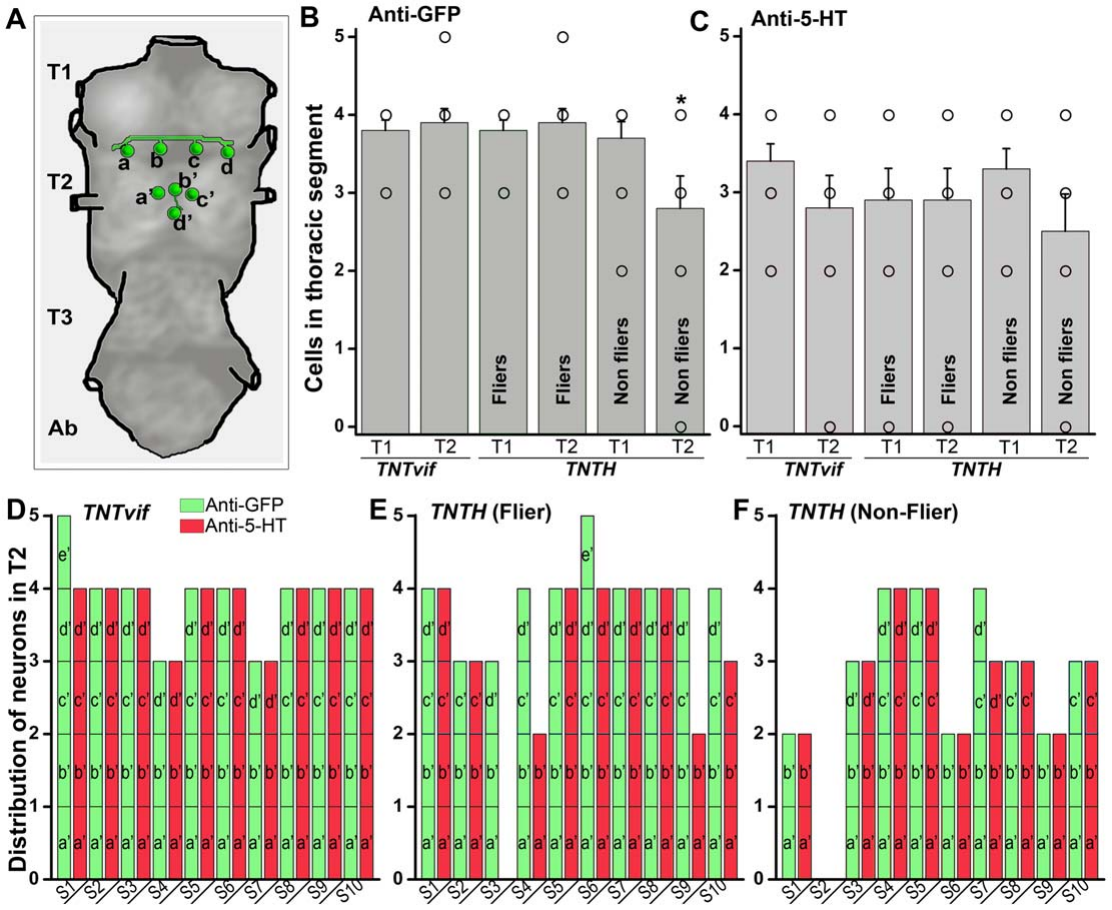
Distribution of serotonergic neurons in second thoracic segment across 10 samples. **A**) Schematic representation of the serotonergic neurons as seen in T1 and T2 region of the thoracic ganglia, showing the average number of T1 and T2 cells. **B**) Number of cells marked by anti-GFP in thoracic ganglia. Non-fliers of the genotype *TRH/TNT* have fewer GFP-positive neurons in T2 as compared with TNTvif controls (what about comparison with fliers also) (*p<0.05; Student's *t* test). **C**) Number of cells marked by anti-GFP in thoracic ganglia. No significant difference is seen with anti-5-HT staining. **D**) TNTvif expression in *TRHGAL4* shows 4 cells, T2a′–d′, in the T2 region. An extra cell, T2e′ is seen in sample S1, which is marked by anti-GFP but not anti-5-HT. In samples S2–S10 equal number of cells were marked by anti-GFP and anti-5-HT staining. **E**) Fliers of the genotype *TRH/TNT* show variation in T2c′,d′ cells, but the variation is not significantly different from TNTvif controls (Fig. 6D). **F**) *TRH/TNT* non-fliers lack T2c′,d′ in sample S1, S6 and S9. Sample S2 lacks all T2a′–d′ cells.

Next, serotonergic neurons in the thoracic segments were quantified, since in principle they were most likely to modulate the flight central pattern generator (CPG) [28]. Variation in the number of dopaminergic and serotonergic cells has been observed in thoracic segments amongst animals of the same genotype [29]. In the first thoracic segment (T1), 4 cells (denoted as a, b, c and d) were observed in nearly all the samples, including non-fliers of the *TRH/TNT* genotype (Fig. 6A–C). The T2 region also had 4 cells, a′, b′, c′ and d′. In controls and *TRH/TNT* fliers, 1/10 flies had a fifth cell in the T2 region marked by anti-GFP, although this extra cell did not counter stain with anti-5-HT (denoted as T2e′) (Fig. 6B). Thus, on an average, there were 4 cells each seen in the T1 and T2 segments of TNTvif controls and TNT fliers with anti GFP (Fig. 7A, B). However, all the GFP positive cells were not always marked by anti-5-HT staining (Fig. 7C). Interestingly, non-fliers among the TNT expressing animals had significantly reduced GFP positive cells in the T2 segment (Fig. 7B) and this trend was also observed in the 5-HT positive cells (Fig. 7C). Because of the observed variation amongst T2 neurons, individual cells were counted in this region and compared across 10 control and 10 *TRH/TNT* animals. In TNTvif controls, TNT fliers and non-fliers, T2a′ and b′ neurons were nearly always present with the exception of one individual in TNT non-fliers (sample 2; Fig. 7D–F) where all four T2 cells were absent. Variation existed in T2c′ and d ‘neurons. Based on cell numbers observed with anti-GFP and anti-5-HT staining, the T2d’ cells were absent in 6/10 individuals of TNT non-fliers (Fig. 7 F), and the T2c′ cells were absent in 4/10 such individuals. Moreover, in the TNT populations, fewer anti-GFP cells were marked by anti-5-HT (Fig. 7E, F) suggesting a reduced level of serotonin. In the abdominal segments of flies expressing either TNT or TNTvif, 7 pairs of GFP-positive cells were observed, all of which were 5-HT negative (Fig. 6).

## Discussion

The importance of aminergic neurons in Drosophila air-puff stimulated flight has been shown previously in the context of IP_3_R signaling and SOCE [8], [30]. However, these data are not straightforward. While *itpr^+^* expression in *DdCGAL4* expressing neurons can rescue flight defects in *itpr* mutants, knock down of the InsP_3_R in the *DdCGAL4* domain by RNAi does not result in any observable flight defects, apart from hyper-excitability of the neural circuit. Interestingly, *itpr* mutant flight defects can also be rescued by *itpr^+^* expression in the *Dilp2GAL4* neuronal domain, which does not overlap with *DdCGAL4* [24]. Thus a possible explanation for the rescue of flight defects in *itpr* mutants by *DdCGAL4* and *Dilp2GAL4* could be non-cell autonomous mechanisms involving in one case neurohormonal release of serotonin and/or dopamine and in the other secreted neurohormones such as the insulin-like peptides. The absence of flight defects in the knock down of InsP_3_R and SOCE components by *TRHGAL4* (Figure 3) as well as the absence of rescue by *TRHGAL4* driven expression of *itpr^+^* (data not shown) suggests that *itpr* mutant flight defects are not derived from intracellular calcium signaling deficits in serotonergic neurons. Increased spontaneous firing from the DLMs upon RNAi mediated silencing of IP_3_R and components of SOCE in the *TRHGAL4* domain suggests that perturbing intracellular calcium homeostasis affects overall activity patterns of the flight circuit, but this change is insufficient for introducing measurable flight deficits.

In locusts, serotonin acts on the fast extensor and flexor tibiae motor neurons and this results in potentiation of synaptic transmission between these neurons, thereby modulating their neuronal properties and synaptic strengths [31]. The partial flight deficit observed in Drosophila by synaptic inhibition of serotonergic neurons could be due to loss or reduction in similar modulatory effects of serotonin on as yet unidentified neurons of the flight circuit. Studies in locusts have also shown that a flight central pattern generator (CPG) residing in the thorax [28], drives the motoneurons and maintains the phase relationship among the motor units of each muscle [3]. Biogenic amines, such as octopamine and tyramine have been shown to modulate the flight CPG in locusts, Manduca and other moths [11], [32]. Though precise components of flight CPG are unknown, it is thought to be activated by a muscarinic cholinergic mechanism in locusts [33]. Because octopaminergic modulation of Drosophila flight CPG has already been shown [12], it is likely that the flight CPG is modulated by multiple neuromodulators including serotonin. Our data suggest that the function of these neuromodulators can be compensated by each other. Temporal blocking of synaptic function in TRH neurons by expressing a temperature sensitive dynamin transgene, *UASShi^ts^* demonstrated a greater requirement for synaptic activity in serotonergic neurons during pupal development, followed by a reduced requirement in adults. In Drosophila, components of indirect flight motoneurons undergo dendritic and axonal remodeling during early pupal stages [34]. In moths, adult flight motor patterns are exhibited during mid-pupal stages [15], [16], [35], indicating that the flight CPG is formed before the mid-pupal stage. Our data support a requirement for synaptic activity in serotonergic neurons during development of the flight CPG. The absence of variation in the numbers of *TRHGAL4* positive but 5-HT negative neurons between fliers and non-fliers indicates that these neurons do not contribute to the flight phenotypes observed. However, at this stage we cannot completely rule out a role for *TRHGAL4* positive neurons that remain 5-HT negative in Drosophila flight.

Loss of serotonergic neurons in the T2 segment by TNT expression suggests that they undergo cell death. Alternately, they may cease to produce serotonin, and their cell fates are re-specified in an activity-dependent manner. Activity-dependent neurotransmitter re-specification has been shown in *Xenopus* larvae. However in *Xenopus*, increased Ca^2+^ spikes reduced the serotonergic cell population in the raphe, a serotonin rich region in the hindbrain [36] while decreased Ca^2+^ spikes, increased the cell population. The spike activity had a converse relationship to the expression of a transcription factor, LmX1b, which is required for the maintenance of serotonin expression in the CNS. Re-specification of neurotransmitters, through altered neuronal activity, can also take place in adults, after synapse formation. This is often triggered by sensory stimuli [36], [37], [38]. Thus the reduced flight in animals where synaptic activity was inhibited in adults could arise either from loss of synaptic activity affecting serotonergic modulation of flight CPG neurons during flight or it could be a consequence of re-specification of serotonergic neurons post-pupal development. This work identifies pupal development in Drosophila as a phase where serotonergic neurons of the flight circuit may be more sensitive to activity-dependent re-modelling. Identification of genes that drive this re-modelling will be of interest.

## References

[1] Gomez TM, Spitzer NC (2000) Regulation of growth cone behavior by calcium: new dynamics to earlier perspectives. J Neurobiol 44: 174–183. 10934320

[2] Spitzer NC (2002) Activity-dependent neuronal differentiation prior to synapse formation: the functions of calcium transients. J Physiol Paris 96: 73–80. 11755785 10.1016/s0928-4257(01)00082-1

[3] Dickinson MH, Tu MS (1997) The Function of Dipteran Flight Muscle. Comparative Biochemistry and Physiology Part A: Physiology 116: 223–238.

[4] Tanouye MA, King DG (1983) Giant Fibre Activation of Direct Flight Muscles in *Drosophila* . Journal of Experimental Biology 105: 241–251.

[5] Engel JE, Wu CF (1996) Altered habituation of an identified escape circuit in Drosophila memory mutants. J Neurosci 16: 3486–3499. 8627381 10.1523/JNEUROSCI.16-10-03486.1996 PMC6579151

[6] Fayyazuddin A, Zaheer MA, Hiesinger PR, Bellen HJ (2006) The nicotinic acetylcholine receptor Dalpha7 is required for an escape behavior in Drosophila. PLoS Biol 4: e63. 16494528 10.1371/journal.pbio.0040063 PMC1382016

[7] Card G, Dickinson MH (2008) Visually mediated motor planning in the escape response of Drosophila. Curr Biol 18: 1300–1307. 18760606 10.1016/j.cub.2008.07.094

[8] Banerjee S, Lee J, Venkatesh K, Wu CF, Hasan G (2004) Loss of flight and associated neuronal rhythmicity in inositol 1,4,5-trisphosphate receptor mutants of Drosophila. J Neurosci 24: 7869–7878. 15356199 10.1523/JNEUROSCI.0656-04.2004 PMC1289272

[9] Sombati S, Hoyle G (1984) Generation of specific behaviors in a locust by local release into neuropil of the natural neuromodulator octopamine. J Neurobiol 15: 481–506. 6097645 10.1002/neu.480150607

[10] Ramirez JM, Pearson KG (1991) Octopaminergic modulation of interneurons in the flight system of the locust. J Neurophysiol 66: 1522–1537. 1765792 10.1152/jn.1991.66.5.1522

[11] Stevenson P, Meuser S (1997) Octopaminergic innervation and modulation of a locust flight steering muscle. J Exp Biol 200: 633–642. 9318358 10.1242/jeb.200.3.633

[12] Brembs B, Christiansen F, Pfluger HJ, Duch C (2007) Flight initiation and maintenance deficits in flies with genetically altered biogenic amine levels. J Neurosci 27: 11122–11131. 17928454 10.1523/JNEUROSCI.2704-07.2007 PMC6672854

[13] Venkiteswaran G, Hasan G (2009) Intracellular Ca2+ signaling and store-operated Ca2+ entry are required in Drosophila neurons for flight. Proc Natl Acad Sci U S A 106: 10326–10331. 19515818 10.1073/pnas.0902982106 PMC2700899

[14] Consoulas C, Restifo LL, Levine RB (2002) Dendritic remodeling and growth of motoneurons during metamorphosis of Drosophila melanogaster. J Neurosci 22: 4906–4917. 12077188 10.1523/JNEUROSCI.22-12-04906.2002 PMC6757714

[15] Vierk R, Duch C, Pfluger HJ (2010) Postembryonic development of centrally generated flight motor patterns in the hawkmoth, Manduca sexta. J Comp Physiol A Neuroethol Sens Neural Behav Physiol 196: 37–50. 19924416 10.1007/s00359-009-0490-z

[16] Kammer AE, Rheuben MB (1976) Adult motor patterns produced by moth pupae during development. J Exp Biol 65: 65–84. 993706 10.1242/jeb.65.1.65

[17] Sweeney ST, Broadie K, Keane J, Niemann H, O'Kane CJ (1995) Targeted expression of tetanus toxin light chain in Drosophila specifically eliminates synaptic transmission and causes behavioral defects. Neuron 14: 341–351. 7857643 10.1016/0896-6273(95)90290-2

[18] Baines RA, Uhler JP, Thompson A, Sweeney ST, Bate M (2001) Altered electrical properties in Drosophila neurons developing without synaptic transmission. J Neurosci 21: 1523–1531. 11222642 10.1523/JNEUROSCI.21-05-01523.2001 PMC6762927

[19] Kitamoto T (2001) Conditional modification of behavior in Drosophila by targeted expression of a temperature-sensitive shibire allele in defined neurons. J Neurobiol 47: 81–92. 11291099 10.1002/neu.1018

[20] Dietzl G, Chen D, Schnorrer F, Su KC, Barinova Y, et al (2007) A genome-wide transgenic RNAi library for conditional gene inactivation in Drosophila. Nature 448: 151–156. 17625558 10.1038/nature05954

[21] Li H, Chaney S, Roberts IJ, Forte M, Hirsh J (2000) Ectopic G-protein expression in dopamine and serotonin neurons blocks cocaine sensitization in Drosophila melanogaster. Curr Biol 10: 211–214. 10704417 10.1016/s0960-9822(00)00340-7

[22] Paradis S, Sweeney ST, Davis GW (2001) Homeostatic control of presynaptic release is triggered by postsynaptic membrane depolarization. Neuron 30: 737–749. 11430807 10.1016/s0896-6273(01)00326-9

[23] White BH, Peabody NC (2009) Neurotrapping: cellular screens to identify the neural substrates of behavior in Drosophila. Front Mol Neurosci 2: 20. 19949456 10.3389/neuro.02.020.2009 PMC2783026

[24] Agrawal N, Venkiteswaran G, Sadaf S, Padmanabhan N, Banerjee S, et al (2010) Inositol 1,4,5-trisphosphate receptor and dSTIM function in Drosophila insulin-producing neurons regulates systemic intracellular calcium homeostasis and flight. J Neurosci 30: 1301–1313. 20107057 10.1523/JNEUROSCI.3668-09.2010 PMC6633787

[25] Valles AM, White K (1988) Serotonin-containing neurons in Drosophila melanogaster: development and distribution. J Comp Neurol 268: 414–428. 3129459 10.1002/cne.902680310

[26] Monastirioti M (1999) Biogenic amine systems in the fruit fly Drosophila melanogaster. Microsc Res Tech 45: 106–121. 10332728 10.1002/(SICI)1097-0029(19990415)45:2<106::AID-JEMT5>3.0.CO;2-3

[27] Alekseyenko OV, Lee C, Kravitz EA (2010) Targeted manipulation of serotonergic neurotransmission affects the escalation of aggression in adult male Drosophila melanogaster. PLoS One 5: e10806. 20520823 10.1371/journal.pone.0010806 PMC2875409

[28] Edwards JS (2006) The central nervous control of insect flight. 1961. J Exp Biol 209: 4411–4413. 17079710 10.1242/jeb.02592

[29] Sykes PA, Norman HS, Condron BG (2004) Variation in serotonergic and dopaminergic neuronal survival in the central nervous system of adult Drosophila. Cell Tissue Res 317: 327–331. 15322910 10.1007/s00441-004-0940-4

[30] Hasan G, Venkiteswaran G (2010) The enigma of store-operated ca-entry in neurons: answers from the Drosophila flight circuit. Front Neural Circuits 4: 10. 20407638 10.3389/fncir.2010.00010 PMC2856631

[31] Parker D (1995) Serotonergic modulation of locust motor neurons. J Neurophysiol 73: 923–932. 7541832 10.1152/jn.1995.73.3.923

[32] Sombati S, Hoyle G (1984) Central nervous sensitization and dishabituation of reflex action in an insect by the neuromodulator octopamine. J Neurobiol 15: 455–480. 6097644 10.1002/neu.480150606

[33] Buhl E, Schildberger K, Stevenson PA (2008) A muscarinic cholinergic mechanism underlies activation of the central pattern generator for locust flight. J Exp Biol 211: 2346–2357. 18587129 10.1242/jeb.017384

[34] Consoulas C, Levine RB, Restifo LL (2005) The steroid hormone-regulated gene Broad Complex is required for dendritic growth of motoneurons during metamorphosis of Drosophila. J Comp Neurol 485: 321–337. 15803508 10.1002/cne.20499

[35] Fenelon VS, Casasnovas B, Simmers J, Meyrand P (1998) Development of rhythmic pattern generators. Curr Opin Neurobiol 8: 705–709. 9914238 10.1016/s0959-4388(98)80111-6

[36] Demarque M, Spitzer NC (2010) Activity-dependent expression of Lmx1b regulates specification of serotonergic neurons modulating swimming behavior. Neuron 67: 321–334. 20670838 10.1016/j.neuron.2010.06.006 PMC2913149

[37] Velazquez-Ulloa NA, Spitzer NC, Dulcis D (2011) Contexts for dopamine specification by calcium spike activity in the CNS. J Neurosci 31: 78–88. 21209192 10.1523/JNEUROSCI.3542-10.2011 PMC3080040

[38] Spitzer NC (2012) Activity-dependent neurotransmitter respecification. Nat Rev Neurosci 13: 94–106. 22251956 10.1038/nrn3154 PMC4352171

